# A computational study on the optimization of transcranial temporal interfering stimulation with high‐definition electrodes using unsupervised neural networks

**DOI:** 10.1002/hbm.26181

**Published:** 2022-12-17

**Authors:** Sangkyu Bahn, Chany Lee, Bo‐Yeong Kang

**Affiliations:** ^1^ Cognitive Science Research Group Korea Brain Research Institute Daegu Republic of Korea; ^2^ School of Convergence Kyungpook National University Daegu Republic of Korea

**Keywords:** noninvasive deep brain stimulation, optimization, transcranial temporal interference stimulation, unsupervised neural network

## Abstract

Transcranial temporal interfering stimulation (tTIS) can focally stimulate deep parts of the brain related to specific functions using beats at two high frequencies that do not individually affect the human brain. However, the complexity and nonlinearity of the simulation limit it in terms of calculation time and optimization precision. We propose a method to quickly optimize the interfering current value of high‐definition electrodes, which can finely stimulate the deep part of the brain, using an unsupervised neural network (USNN) for tTIS. We linked a network that generates the values of electrode currents to another network, which is constructed to compute the interference exposure, for optimization by comparing the generated stimulus with the target stimulus. Further, a computational study was conducted using 16 realistic head models. We also compared tTIS with transcranial alternating current stimulation (tACS), in terms of performance and characteristics. The proposed method generated the strongest stimulation at the target, even when targeting deep areas or performing multi‐target stimulation. The high‐definition tTISl was less affected than tACS by target depth, and mis‐stimulation was reduced compared with the case of using two‐pair inferential stimulation in deep region. The optimization of the electrode currents for the target stimulus could be performed in 3 min. Using the proposed USNN for tTIS, we demonstrated that the electrode currents of tTIS can be optimized quickly and accurately. Moreover, we confirmed the possibility of precisely stimulating the deep parts of the brain via transcranial electrical stimulation.

## Graphical abstract

We proposed an unsupervised neural network to optimize electrode current for tTIS. Simulations using 69 electrodes for 16 head models were tested. Optimization in a deep brain region could be performed in 3 min. Simultaneous stimulation on two regions was well optimized.

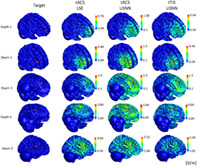

## 1 INTRODUCTION

Transcranial electric stimulation (tES), a noninvasive method for stimulating the brain by applying an electric current to the scalp, has gained attention because of its safety (Nitsche & Paulus, 2000). This method is effective in enhancing brain cognitive function or as an alternative to pharmacology in the treatment of psychopathy (Darkow et al., 2017; Fregni et al., 2006; Sauvaget et al., 2015; Soler et al., 2010). In classic tES, a large sponge is attached near the area to be stimulated (Siebner, 2004). Numerical analysis using techniques such as the finite element and boundary element methods can be performed to analyze the electric fields of the brain. An increased number of electrodes configured, for example, according to the international 10–10 system, can be used to stimulate the desired area more precisely (Edwards et al., 2013; Sreeraj et al., 2018).

The transcranial alternating current stimulation (tACS) method, which exposes specific brain regions through alternating current stimuli, is also used for electrical brain stimulation (Antal & Paulus, 2013; Zaehle et al., 2010). However, tDCS and tACS, which inject current into the scalp, have difficulty stimulating only the deep regions of the brain because a large electric field tends to form around the electrodes (Bai et al., 2013; Im et al., 2008). As an alternative, a method for obtaining only a mediating effect by stimulating the shallow region, which is speculated to be connected to the deep region, is used, rather than directly stimulating the deep region (Gomez‐Tames et al., 2020). To overcome this limitation, transcranial temporal interfering stimulation (tTIS), which uses the beat caused by the interference of two different frequencies, has been proposed to modulate the deep region focally (Grossman et al., 2017).

Numerous studies on the tTIS method, which analyzes the biological phenomena occurring in the brain, have been conducted (Esmaeilpour et al., 2021; Grossman et al., 2018; Huang & Parra, 2019; Mirzakhalili et al., 2020; Rampersad et al., 2019; Sunshine et al., 2021). In addition, a device has been proposed for multichannel tTIS and corresponding in vivo experiments conducted; however, the optimization applied in the study was based on a sphere model, which limited the scope of the study to shallow brain targets (Song et al., 2021).

In contrast to tDCS and tACS, which enable linear combinations, the tTIS‐relevant modulation distribution is nonlinear, and optimization of the electrode currents is challenging. In several studies, optimization has been performed using a limited number of electrode sets, such as two pairs (Cao & Grover, 2020; Honarbakhsh & Mohammadzadeh, 2020; Lee et al., 2020; Rampersad et al., 2019; Su et al., 2021; Xiao et al., 2019). It is efficient and realistic arrangement in terms of hardware design, clinical experiments, and error sensitivity. However, even in such a simple arrangement, its optimization is computationally challenging, and the location and current of the electrodes were determined empirically, by comparing several combinations (Lee et al., 2020) or by adjusting the location gradually (Rampersad et al., 2019).

Huang et al. (2020) attempted to go beyond two pairs of electrodes to optimize deep stimulation targeting, and used neck part electrodes in addition to the international 10–10 system. Further, as an optimization method, sequential quadratic programming (SQP) (Brayton et al., 1979) has been utilized for local search to concentrate the stimulus by gradually releasing the power constraint. However, this method requires running the SQP function several times for each optimization, which adds to its numerical cost and runtime.

In this study, the electrode currents of tTIS are optimized using unsupervised neural networks (USNNs). Neural networks are an approach that solves problems by imitating the connections among neurons in the human brain (Goodfellow et al., 2016; Kröse et al., 1993). Recently, this method has received considerable attention in various fields, with advances in hardware such as graphics processing unit computation (Chen et al., 2014; Cui et al., 2016) and the availability of large data.

The mainstream neural network adopts supervised training using ground truth data. However, the problem of determining the electrode currents that stimulate the target region cannot be resolved until the optimization process is performed. Therefore, a USNN was considered to address this problem. The main idea of this approach is to fix specific formulas in the neural network of the feed‐forward structure. The neural network is trained in a direction that minimizes the output of the network when an input is provided without a dataset. In this process, a specific connection weight corresponding to the actual output is obtained. This approach is frequently used to solve nonlinear problems (Monterola & Saloma, 2001; Raja et al., 2014) and differential equations (Parisi et al., 2003; Shirvany et al., 2009; Yadav et al., 2015).

The basic concept of this method is that a neural network generates a set of electrode currents, and the stimulus is obtained using a fixed network converted from a stimulus formula. The set of electrode currents is optimized by comparing the calculated stimulus with the target stimulus.

## 2 MATERIALS AND METHODS

### 2.1 Realistic head model

**FIGURE 1:**
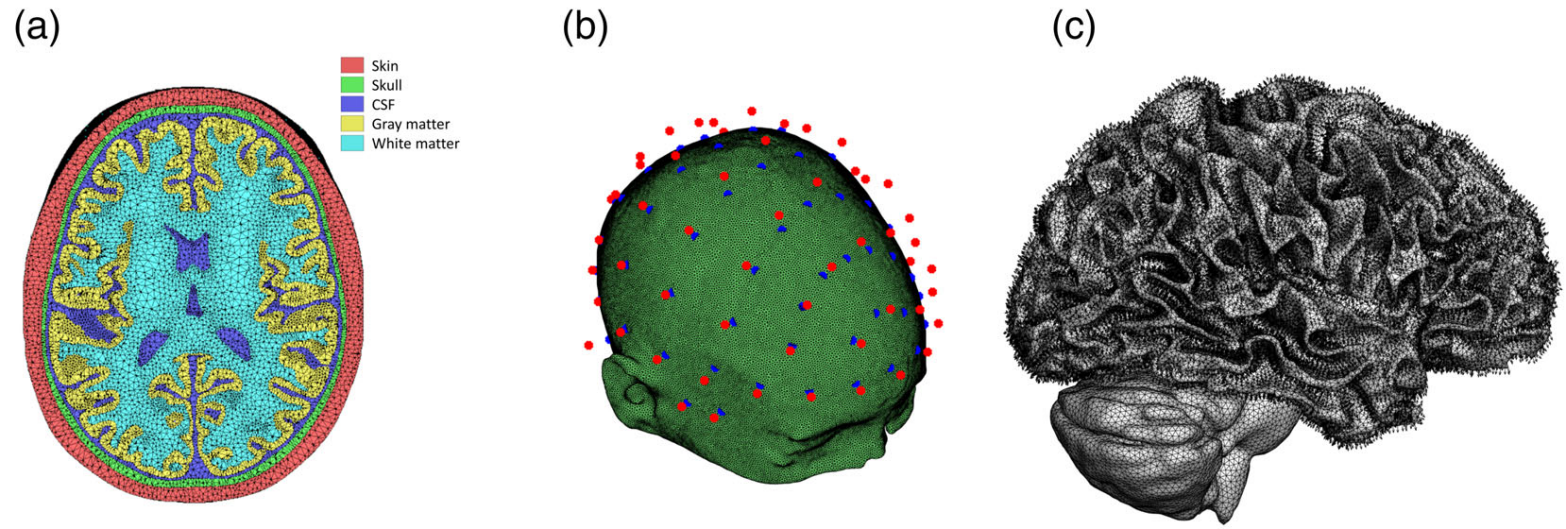
(a) Five‐layer tetrahedral meshed finite element model consisting of the skin, skull, cerebrospinal fluid, gray matter, and white matter. (b) Electrode placement. Red dots indicate the placement positions based on the 10–10 system on the ellipsoid, and blue dots are the positions projected to the nearest scalp. (c) Normal unit vector plots on all nodes at the interface of white and gray matter

The head models used in the simulation are derived from “ICBM” data (Van Essen et al., 2012) and averaged pediatric data (Fonov et al., 2009; Fonov et al., 2011), which are open data of brain magnetic resonance imaging (MRI) images. These MRI data were reconstructed into a five‐layer finite element model comprising the skin, skull, cerebrospinal fluid, gray matter, and white matter using a public segmentation tool named SIMNIBS (Saturnino, Puonti, et al., 2019) (Figure 1a). The 16 head models were discretized into 3,615,803 ± 144,165 tetrahedrons with a volume of 0.861 ± 0.858 mm^3^, and the assigned electrical conductivities were 0.465, 0.010, 1.654, 0.276, and 0.126 S/m at the skin, skull, cerebrospinal fluid, and gray and white matter (Wagner et al., 2008), respectively.

Based on the international 10–10 system, 69 electrodes with a diameter of 1 cm were used for stimulation. To attach these electrodes to the head, as shown in Figure 1b, the electrode was placed on an elliptical hemisphere that approximated the head and then projected to the nearest position of the head. In this electrode system, the electrodes were assumed to produce two types of frequencies (Huang et al., 2020).

The normal direction of the cerebral cortex was used to extract the stimulation‐relevant exposure component because anatomical information indicates that cortical pyramidal neurons extend in the perpendicular direction (Kuo et al., 2013; Lee et al., 2017). Since the outer boundary of gray matter cannot express narrow wrinkles at low MRI resolutions, the outer boundary of white matter, where this issue is not prominent, is targeted. At the white matter boundary, each node is connected to its adjacent nodes by the edges of the triangle. A plane regressed from these adjacent nodes can be determined, and its normal vector is defined as the normal direction of each boundary node (Figure 1c). In this study, the cerebral cortex with a well‐known direction of stimulation was used, and other tissues (e.g., the striatum, hippocampus, or thalamus sub‐regions) could be added if there was a verified location and required direction of stimulation.

### 2.2 Mathematical formulation of the stimulation

To calculate the electric potential *V* generated by the current injected through the electrodes, the Laplace equation $-\nabla ∙\left(\sigma \nabla V\right)=0$ (σ: electric conductivity) was solved using the finite element method with linear approximation (Lee et al., 2017). The electric field, which is the negative gradient of the electric potential, was then obtained. The total normal component of the electric field on the interface between the gray and white matter in this study, can be computed using the following matrix form (Dmochowski et al., 2011):

(1) $$E_{\text{total}}=\left(\begin{array}{c} \begin{array}{c} E_{1}\\ \vdots \\ E_{n} \end{array}\\ \vdots \\ E_{N} \end{array}\right)=\left(\begin{array}{cc} \begin{array}{ccc} E_{1,2} & \cdots & E_{1,m}\\ \vdots & \ddots & \cdots \\ E_{n,2} & \cdots & E_{n,m} \end{array} & \begin{array}{cc} \cdots & E_{1,M}\\ \ddots & \vdots \\ \cdots & E_{n,M} \end{array}\\ \begin{array}{ccc} \vdots & \ddots & \cdots \\ E_{N,2} & \cdots & E_{N,m} \end{array} & \begin{array}{cc} \ddots & \vdots \\ \cdots & E_{N,M} \end{array} \end{array}\right)\;\left(\begin{array}{c} \begin{array}{c} I_{2}\\ \vdots \\ I_{m} \end{array}\\ \vdots \\ I_{M} \end{array}\right)=\mathrm{EI},$$

where $I_{m}$ is the electric current between the *m*th electrode and the reference electrode (chosen in this study as the first electrode), $E_{n,m}$ is the normal component of electric field at the *n*th node generated by the unit current between the *m*th electrode and the reference electrode, and $E_{n}$ is the total nomal component of electric field at the *n*th node. In this study, we considered the normal component of the electric field on the cortical surface.

$E_{\text{target}}$ is the ideal normal component of electric field distribution of the entire location to be made through the electrode current, and generally a vector, in which the target locations have a value of one and the others have a value of zero, is used. The electrode currents, which optimize the amplitude of the electric field of a single frequency to the target, can be calculated by multiplying both sides of Equation 1 with the pseudo‐inverse of *E*. This method is called the least squares estimation (LSE), and a set of currents with least square errors can be found for tACS problems, modeled in linear combinations (Dmochowski et al., 2011; Lee et al., 2017).

(2) $$I_{\mathrm{LSE}}=E^{-1}E_{\text{target}}.$$

### 2.3 Modulation of tTIS

When two different high‐frequency currents are injected on both sides of the head, a beat with an interference frequency corresponding to the difference between the two frequencies is generated. The frequencies of the injected current do not individually affect the human brain (due to their unphysiological high frequency), while the brain responds to their combined modulation at the difference frequency. The amplitude of the beat can be stronger in the deep region than in the shallow region. Naturally, the modulation of the brain is not determined only by the size of the beats. The sensitivity to the beat amplitude varies depending on the type of organization; it is not modulated in proportion to the beat amplitude. However, this property of the entire brain has not yet been verified. To simplify the computational study, it is assumed that the modulation is affected only by the amplitude of the beats.

The beat modulation equation is expressed as follows:

(3) $$\mathrm{Mod}\left(n\right)=\left|E_{n}^{f_{1}}\right|+\left|E_{n}^{f_{2}}\right|-\left|\left|E_{n}^{f_{1}}\right|-\left|E_{n}^{f_{2}}\right|\right|=2\;\min\left(E_{n}^{f_{1}},E_{n}^{f_{2}}\right),$$

where $E_{n}^{f}$ is the peak value of the electric field of the alternating current of frequency *f* at location *n*. In this study, the peak‐to‐peak signal of the electric field was used as the output value indicating the modulation of tACS. However, because tACS and tTIS have different stimulation mechanisms, these two magnitudes cannot be compared equally. Therefore, a direct comparison of magnitude was avoided, and comparisons of the accuracy of targeting and concentration were conducted.

Beat modulation is a nonlinear combination of electrode currents at different frequencies. Therefore, the LSE method, which determines the optimal current directly in the linear system by multiplying by the pseudo‐inverse, cannot be applied to optimize them. To optimize the electrode current in a nonlinear relation with modulation, a different approach for optimization, such as USNN, is required.

### 2.4 Structure of USNN

**FIGURE 2:**
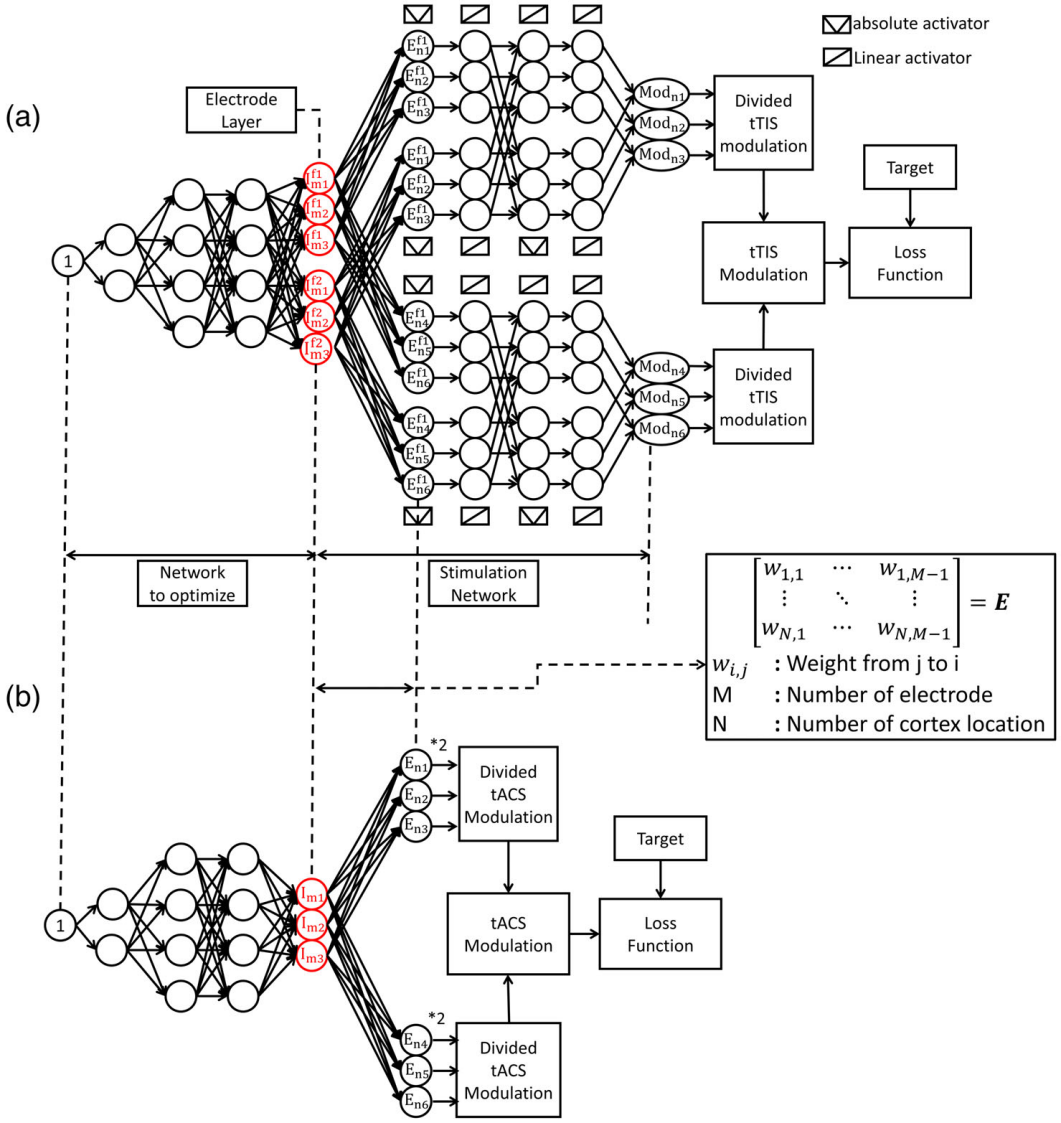
Architecture of USNN for electrode current optimization of (a) tTIS and (b) tACS

Figure 2a shows the tTIS optimization architecture. In this unsupervised training network, no input data are provided, and the unit constant replaces the input. The nodes passing through the layers from the unit constant are fully connected to the electrode layer.

The electrode layer has twice as many nodes as the number of electrodes (reflecting their contribution at two different frequencies). The goal is to optimize the electrode currents that stimulate the target position focally by training the weights of the connections. Each node is connected by a rectified linear unit (ReLU) activation function (Hahnloser et al., 2000), and a normalization layer (Ba et al., 2016) is inserted after all hidden layers to reduce the influence of the initial value and ensure stable convergence. The stimulation network is connected next to the electrode layer to determine the modulation of the interference.

The electric field in the head can be obtained by a linear combination of the reference electric field and currents for each electrode, as shown in Equation 1. Therefore, the fully connected connection between the electrode nodes of a specific frequency and the cortex location nodes is set as matrix *E* in Equation 1. An electric field can be generated at each frequency using these connections. Because many locations exist in the cortex, they can be divided into appropriate sizes. To obtain the modulation of tTIS from the electric field amplitude for each frequency, a custom activation function that returns the absolute value of the input is declared and the electric field layers are connected as given in the formula in sequence. In this study, the first term of Equation 3 is used, and the second term can also be used in the implementation process. The entire network that calculates the modulation from the electrode has a fixed weight that cannot be trained. The obtained modulation is compared with the given target and back‐propagated to train the hidden layers to determine the electrode currents. In the case of tACS using a single frequency, the modulation is defined as twice the amplitude of the electric field and can be obtained directly after the electrode layer, as shown in Figure 2b.

This is the basic concept of the USNN for tTIS. The neural network has a formula for obtaining the stimulation, which is modeled from a reference electric field matrix to determine the modulation of the beats. This enables the neural network to select the appropriate electrode currents from a set of random electrode currents with repeated backpropagation that generates a modulation close to the target. The hidden layers are added to solve the problem in various ways because the method for finding the optimal solution by only gradually changing the electrode currents can easily fall into local minima.

### 2.5 Loss function

Generally, the sum of the squared errors is used to quantify the difference because the loss function returns the difference along with the correct answer, which aims to reduce the error in the neural network. In the brain stimulation problem, the target‐segmented binary vector can be the correct answer in the LSE method. However, the target has a negligible area compared to the entire brain, and the effect of the target's concentration on the result is also small, resulting in a slow convergence rate in the slope descent. For the target‐oriented approach, the three intuitive factors related to successful stimulation are defined as ratios.

The loss function is expressed as the product of three ratios as follows:

(4) $$\text{Loss}=\frac{\mathrm{MR}}{\mathrm{PR}∙\mathrm{CR}}.$$

The peak ratio (PR) is the ratio of the peak modulation of the target to the peak modulation of the non‐target area. Thus, the largest modulation is applied to the target location.

(5) $$\mathrm{PR}=\frac{\max_{i\in \text{Target}}\mathrm{Mod}\left(i\right)}{\max_{i\in \text{Non}-\text{target}}\mathrm{Mod}\left(i\right)}.$$

The concentration ratio (CR) is the ratio of the modulation density of the target to the average modulation density, indicating how much of the modulation is concentrated at the target position compared with the average modulation density. In principle, to express the density of the normal interference component at the gray‐white matter boundary, modulation should be calculated for each element and appropriately weighted according to the area of the triangle. However, simplified calculations, the “Area” was approximated as the number of nodes in a region assuming that the size of elements was relatively uniform. The unit of the denominator and numerator is not the same as density, however, a ratio of density can be obtained, equally.

(6) $$\mathrm{CR}=\frac{\left(\frac{\sum_{i}^{\text{Target}}\mathrm{Mod}\left(i\right)}{{\text{Area}}_{\text{Target}}}\right)}{\left(\frac{\sum_{i}^{\text{Total}}\mathrm{Mod}\left(i\right)}{{\text{Area}}_{\text{Total}}}\right)}.$$

The mis‐stimulation ratio (MR) is the ratio of the mis‐stimulated area to the target area. Here, mis‐stimulation is defined as the position where the modulation is larger than the average modulation of the target.

(7) $$\mathrm{MR}=\frac{{\text{Area}}_{\text{Misstimulated}}}{{\text{Area}}_{\text{Target}}}.$$

The Adam optimizer (Jais et al., 2019; Kingma & Ba, 2017), which optimizes the weights of the network, is based on gradient descent. In this method, the objective function must be continuous and differentiable. Therefore, the mis‐stimulation area was approximated using a sigmoid function centered on the average value (Kim et al., 1993).

(8) $${\text{Area}}_{\text{Misstimulated}}\approx \sum_{i}^{\text{Non}-\text{target}}\frac{1}{1+e^{-\left(\mathrm{Mod}\left(i\right)-\underset{j\in \text{target}}{\text{mean}}\mathrm{Mod}\left(j\right)\right)}}.$$

Naturally, the three factors being multiplied have different ranges, and multiplying them is arbitrary. Proper weighting and renormalization through repeated testing can lead to improved performance. Similarly, the basic sigmoid function of Equation 8 was also chosen arbitrarily and has the potential to be adjusted.

### 2.6 Current normalization

Because the loss function is composed of relative ratios, the electrode currents returned by the proposed USNN must be normalized to achieve values with an appropriate magnitude. Generally, tES limits the magnitude of the electrode current to ensure safety. Many studies have limited the flow of currents to less than 2 mA from each electrode (Huang et al., 2020; Lee et al., 2020; Rampersad et al., 2019) and, in some cases, the sum of the anodal currents is restricted to 4 mA (Electrode: Neuroelectrics User Manual, 2018; Starstim: Neuroelectrics User Manual, 2018). In this study, the modulation magnitude was calculated by applying both of these limitations, and the normalization equation is expressed as follows:

(9) $$I_{1}=I_{0}/\max\left(\left|I_{0}\right|\right)∙2\mathrm{mA},$$

(10) $$\left\langle \begin{array}{c} I=I_{1}\;\left(\mathrm{sum}\left(\left|I_{1}\right|\right)\leq 8\mathrm{mA}\right)\\ I=I_{1}/\mathrm{sum}\left(\left|I_{1}\right|\right)∙8\;\left(\mathrm{sum}\left(\left|I_{1}\right|\right)>8\mathrm{mA}\right) \end{array}\right..$$

In this equation, $I_{0}$ is a current vector generated from the electrode layer of a neural network, and $I_{1}$ is a current vector normalized such that the maximum current of the electrode does not exceed 2 mA. The sum of the currents in all electrodes is zero, and the sum of the anodal currents is equal to the sum of the cathodal currents. Therefore, *I* is a current vector normalized such that the sum of the absolute values of currents is less than 8 mA, which means that the sum of the anodal current is less than 4 mA.

### 2.7 Details of the computational study

#### 2.7.1 Analysis by depth

**FIGURE 3:**
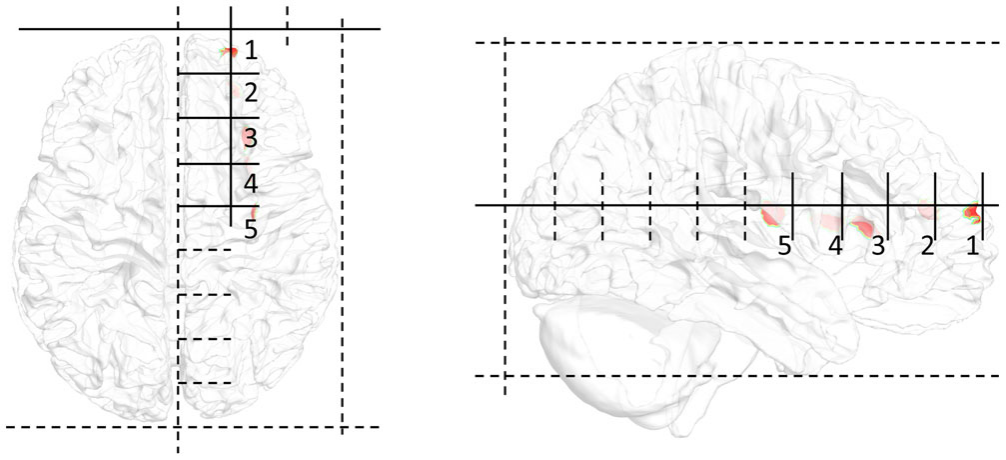
Schematic of the position of the five depth indices. The shallow part has an index number of 1, and the deepest part has an index number of 5

To verify the performance of the proposed method, the changes in the results as a function of depth were analyzed. As shown in Figure 3, by dividing the front and rear endpoints of the head into nine equal parts on the vertical axis, the target positions were determined by entering from the front. The horizontal axis represents the position translated by dividing the right brain into three from the left, and the height is half that of the cerebrum. Therefore, the position was moved to a deeper location along the depth index. Each target consisted of 50 nodes in the cortex closest to the selected location. These five tissues were selected considering only the depth and were statistically tested on 16 head models; however, there are uncertainties due to inter‐subject variability and tissue properties. All LSE and USNN results were compared with those of single‐frequency tACS.

#### 2.7.2 Multi‐stimulation

**FIGURE 4:**
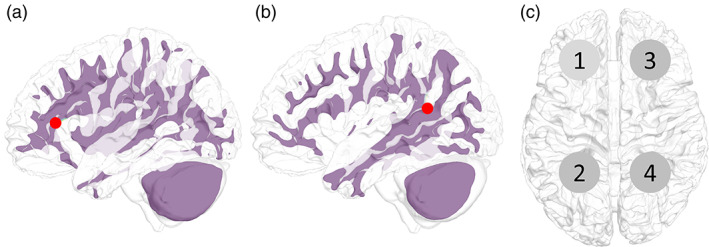
(a) Examples of positions corresponding to position indices 1 and 3 in multi‐stimulation. Folded part of the prefrontal cortex. (b) Examples of positions corresponding to position indices 2 and 4 in multi‐stimulation. This position is the narrow and deep folds on the upper and posterior sides of the brain. (c) Index of multi‐stimulation positions

The deep region for multi‐stimulation was the folded region in the prefrontal cortex (Figure 4a) and the deep wrinkles in the vicinity of the intersection of the parietal, temporal, and occipital lobes (Figure 4b). Multi‐stimulation was tested for pairs 1–2, 1–3, and 1–4 (Figure 4), and the performance evaluation of the two targets in each pair was conducted separately by dividing the brain into two parts. In the pairs 1–2 and 1–3, the brain was divided into left and right hemispheres, and in pair 1–2, it was divided into anterior and posterior hemispheres by a plane at the center. All LSE and USNN results were compared with those of the single‐frequency tACS.

#### 2.7.3 Comparison with existing interference stimulations

Many previous tTIS studies have used two‐pair electrode system to implement tTIS (Cao & Grover, 2020; Honarbakhsh & Mohammadzadeh, 2020; Lee et al., 2020; Rampersad et al., 2019; Su et al., 2021; Xiao et al., 2019). The two‐pair electrode system comprised a set consisting of an anode and a cathode for each frequency, facilitating the determination of the optimal current for position combinations. In studies using a two‐pair electrode system, the optimal currents were calculated for many candidate position combinations, and the best values were selected. The candidate position combinations are obtained by either selecting among various combinations in the 10–10 systems (Lee et al., 2020), or by changing the positions gradually (Rampersad et al., 2019). However, the performance of these arrangements is poor owing to the limited number of electrodes.

In this study, we analyzed the performance improvement compared to a two‐pair electrode system using HD electrode systems. For comparison, a genetic algorithm (GA) (Houck et al., 1995; Kang et al., 2014; Miao et al., 2018) (Appendix A) was used only to obtain a stable solution for the two‐pair electrode system. In the GA, 1000 individuals have chromosomes comprising the position index and electrode current pair for each frequency, and individuals with a high evaluation are selected through crossover, mutation, and elite conservation. A comparison with the two‐pair interfering stimuli was performed in the insular region (Isnard et al., 2011). This comparison was conducted for only one head model (ICBM UTHC 1088 766), and the GA selected the highest value by performing 100 iterations over 1000 generations.

Furthermore, the proposed method was compared with Huang's method (Huang et al., 2020) using an HD electrode system with the same target. The head model, electrode system, finite element analysis, and current constraints were implemented in the same manner as the proposed model, except for the optimization method. This method determines the current combination that maximizes the modulation of the target point by limiting the total modulation power of the nontarget region through the SQP algorithm. The search was repeated while gradually releasing the power constraint, and an appropriate solution was selected. The power constraint was divided into 16 stages from 10^−3^ to 10^12^ times of the total power of the LSE result, and the tACS optimization result obtained using this method was used as the initial value.

The comparison with the existing tTIS methods mentioned above is not fair for evaluating the performance of the optimization algorithm. The two‐pair electrode system has a different parameter space from that of the HD electrode system, and Huang's method has a different objective function for optimization. Therefore, the optimization performance of the USNN was verified through comparison with the GA using the same parameter space and the same objective function. The chromosome of this GA consisted of a list of all electrode currents for two frequencies, and the genetic operation was performed equally in the case of the two‐pair electrode system. A total of 50 tests were conducted, in which a population of 1000 evolved over 10,000 generations.

#### 2.7.4 Study environment

All developments and simulations were performed on a computer with an Intel i9‐9900k CPU, 64 GB RAM, and NVIDIA GeForce RTX 2070 Super GPU. Realistic head modeling and result analysis were performed using MATLAB R2019b, and the finite element analysis of the stimulation was performed via Fortran in Visual Studio 2017. The neural network was developed using TensorFlow 2.3.0, and Keras 2.4.0 in Python 3.7.6.

## 3 RESULTS

### 3.1 Analysis by depth

**FIGURE 5:**
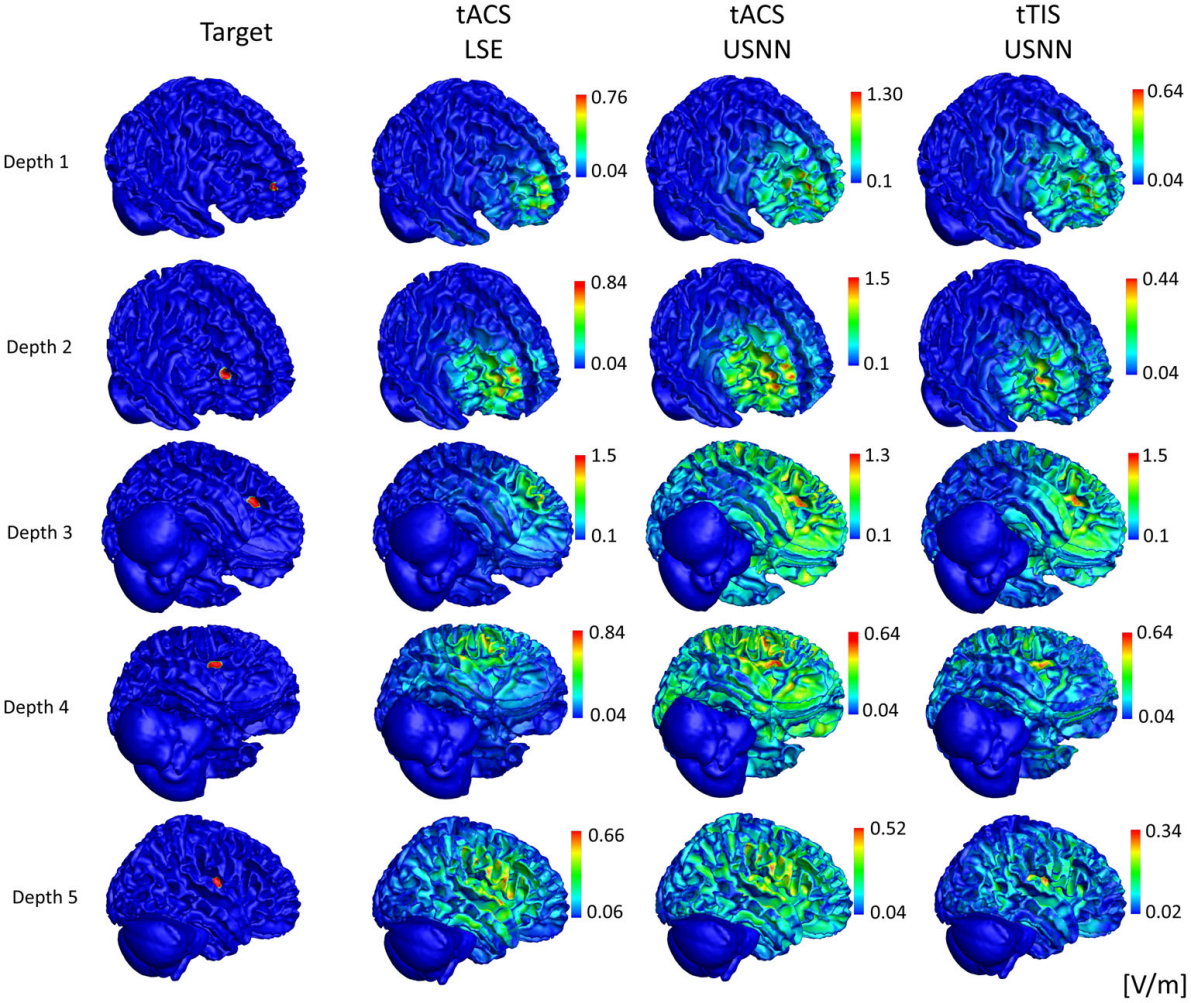
Visual comparison of the modulation generated by the three methods (optimization for tACS by LSE, and for tACS and tTIS by the proposed neural networks) according to depth

**FIGURE 6:**
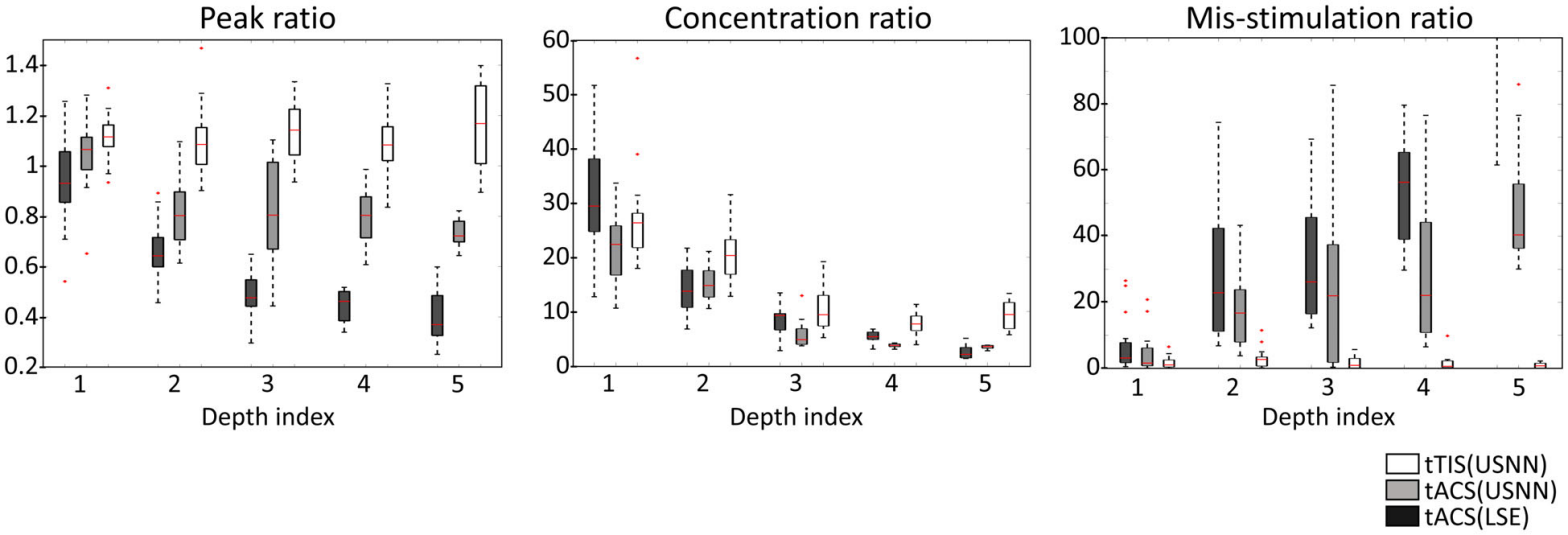
Boxplots of 16 head model results for the three methods according to depth in terms of peak ratio, focal ratio, and mis‐stimulation ratio

Figure 5 shows the results for depth, as a surface plot, on a head model of the optimization using LSE and neural networks for tACS and tTIS. In the case of tACS, the results of neural networks with relatively large stimuli were applied to the target compared with the results of the LSE; however, a slight oscillation can be observed over a wide area. In the case of tTIS, accurate and focused stimulation was performed, even in deep areas. This is also shown in the boxplot of the whole brain model in Figure 6. In contrast to tACS, where the peak and mis‐stimulation ratios worsened with depth, tTIS remained stable. However, in terms of the concentration ratio, tTIS also worsened according to depth but was always better than tACS. The peak value of the modulation of the target using the LSE was lower for all depth indices than for the others. The optimization of USNN was performed for 1000 epochs, which took ~3 min.

### 3.2 Multi‐region stimulation

**FIGURE 7:**
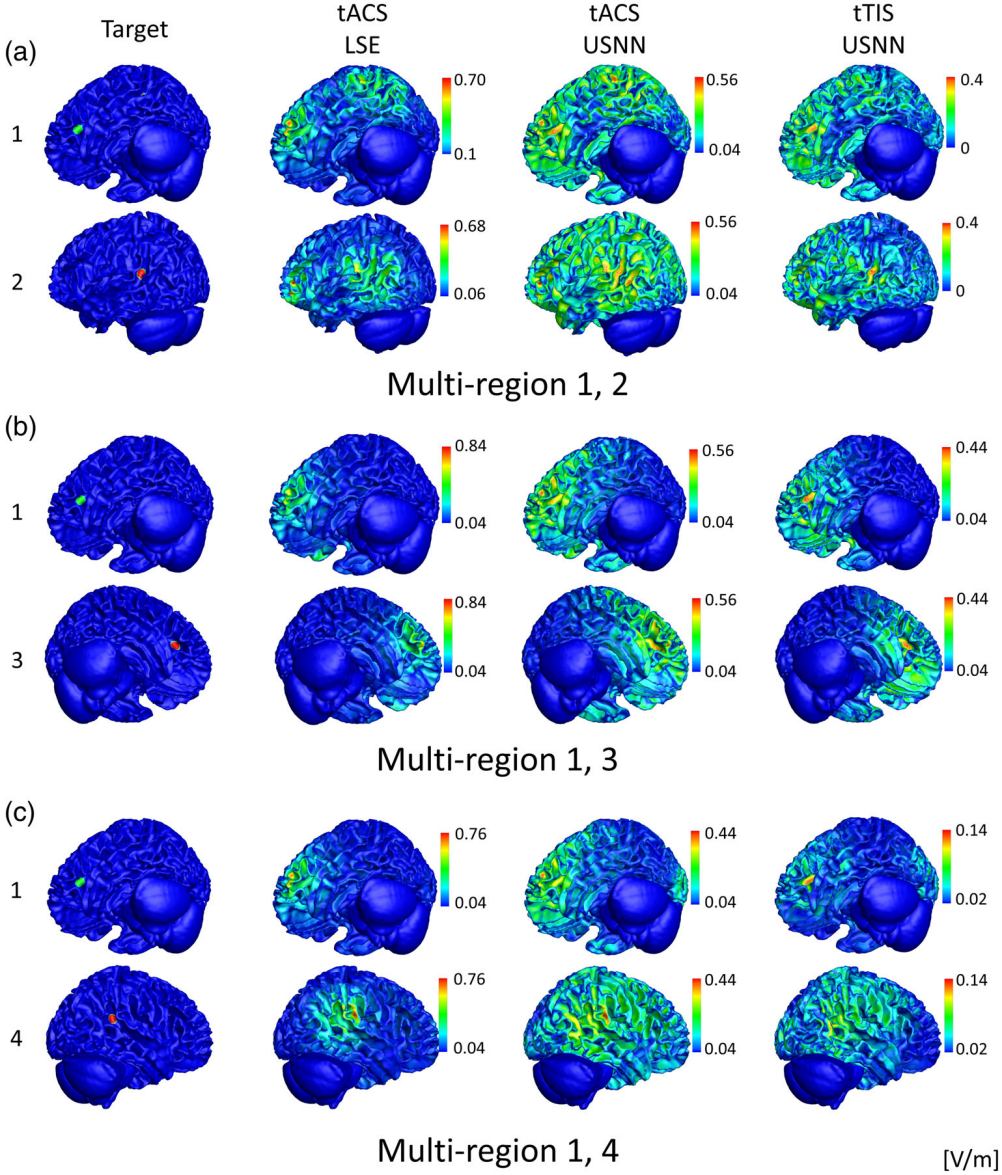
Visual comparison of the modulation generated by the three methods. Optimization (a) by LSE for tACS and by the proposed neural networks for (b) tACS and (c) tTIS used for multi‐region stimulation

**FIGURE 8:**
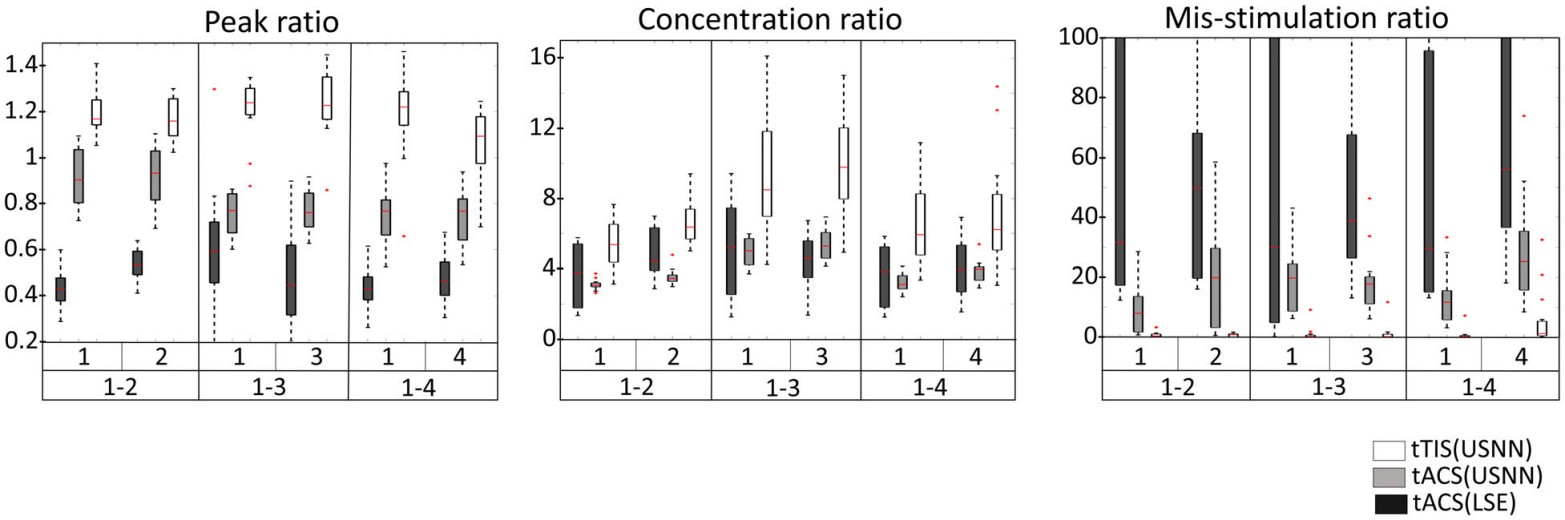
Boxplots of 16 head model results for the three methods used for multi‐stimulation in terms of peak ratio, focal ratio, and mis‐stimulation ratio

Figure 7 shows the results of optimization of multi‐stimulation using LSE and neural networks for tACS and tTIS on a head model as a surface plot. Similar to the test for depth, when tACS is optimized by neural networks, the targets are stimulated more strongly than in the optimization performed by LSE; however, weak modulation is formed over a wide area. This phenomenon is also observed in tTIS, but is less severe than in tACS. In this head model, tTIS failed to stimulate area No. 4 when multiple regions 1 and 4 were stimulated. Figure 8 shows that the quartile is less than one for the peak ratio of area No. 4 on the 1–4 multi‐stimulation. In this case, in addition to the peak ratio, other evaluation ratios showed poor results. However, even including this area, tTIS shows superior accuracy compared with tACS in multi‐region stimuli. The optimization of USNN was performed for 2000 epochs, which took ~6 min.

### 3.3 Comparison with existing interference stimulations

**FIGURE 9:**
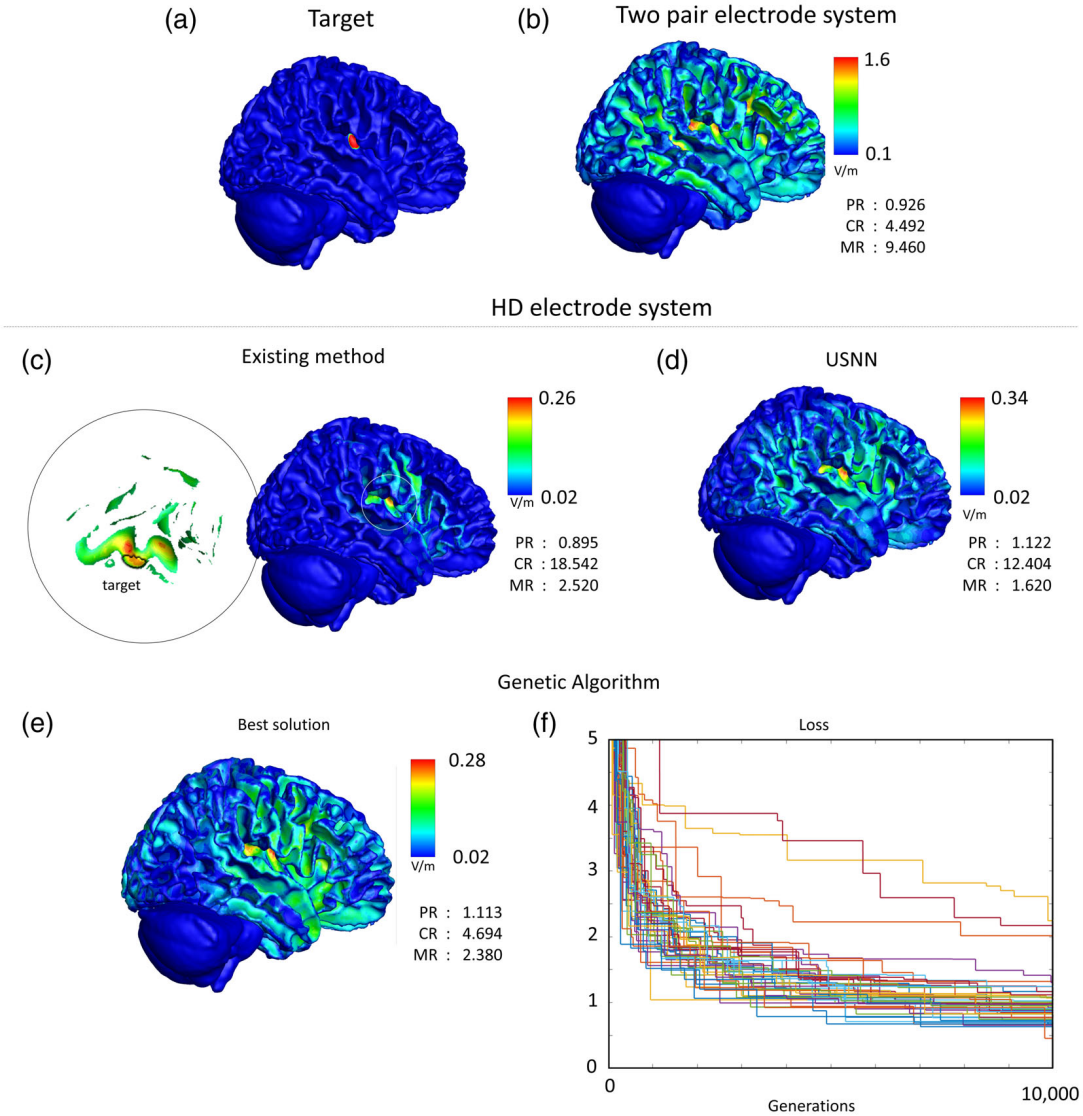
Comparison of optimization results. (a) Insular region used as the target for optimization. (b–e) Visual plot of optimized results of the two pair electrode system via GA (b), HD electrode system through existing method (c), proposed USNN (d), and GA (e). (f) Loss values generated using GA results

Figure 9 shows a comparison with other methods in interfering stimulation results on the insular area in a head model. In the case of two‐pair electrodes, when the GA was repeated 100 times for 1000 generations, 17 equivalent results had the minimum loss value. This result was used as the optimal result for the two‐pair electrode system in this study. In the case of the two pairs, the concentration was much lower than that of the HD methods, and the strongest modulation was within the target (PR <1). The Huang method showed the highest concentration among the three methods; however, targeting was unsuccessful because most of the total power was concentrated on points outside the target area. In the case of USNN, overall, the performance was better than that of the two‐pair stimulation. Although the concentration was lower than that of Huang's method, the strongest modulation was within the target (PR <1). Huang's method took 3 h to optimize, whereas the USNN method took 3 min.

The GA evaluation value of the HD electrode system used to compare the optimization performance with USNN was that of the best individual after 10,000 generations. The loss of the best object out of the population of 1000 in each test can be checked in the lower right corner of Figure 9, and the visual plot of the best object out of the 50 tests is shown in the left corner of Figure 9. Whereas USNN called the evaluation function 1000 times, GA called it 10 million times; however, the best result of all the GA tests had a significantly lower CR (4.694) than that of USNN (12.404).

## 4 DISCUSSION

In this study, we proposed a method for optimizing the current values of HD electrodes through USNN to implement tTIS for stimulating the deep regions of the brain. To evaluate the performance of this method, we constructed intuitive visual plots with peak, concentration, and mis‐stimulation ratios, which are the evaluation metrics. The tests for depth and multi‐stimulation were compared with the results of two types of tACS for two purposes: to analyze the performance characteristics and limitations of tTIS for depth and complex stimulation (multi‐stimulation) compared with tACS, and to analyze the performance improvement of the proposed USNN method compared with the general LSE method in the tACS problem. Because the LSE methods have different objective functions, it cannot be a fair comparison of the optimization performance with USNN. USNN can have an objective function in which the characteristics of a successful stimulus are considered. In this comparison, the ability of the USNN method to better reflects the objective compared to the LSE method, which approximate the ideal modulation distribution in an alternative way. These comparisons were based on 16 realistic head models. This is a large number compared with the number of models (less than three) employed in previous studies; however, it is still a small number for statistical analysis. The variability between models is reflected in the bars in the boxplot (Figures 6 and 8). However, it is important to be aware that these bars only capture anatomical variability and not the general modeling uncertainly resulting from segmentation, discretization, convergence, tissue properties variability, and uncertainty, electrode contact model, and so forth.

The performance of the peak ratio and the mis‐stimulation ratio of both the LSE and neural network results in a single frequency gradually decreasing as the depth increases (Figure 6). The result of a single frequency is a linear combination of the results of the reference electric field for each electrode (Bai et al., 2013; Im et al., 2008). Because all the reference electric fields tend to have a large amplitude in the shallow part near the electrode, the results are naturally poor in the deep region. However, even for a single frequency, when the objective function is used in the proposed method, the performance can be improved to some extent. In the results of tACS using USNN, the concentration was poor, but a stronger modulation was generated in the target position compared with other areas. Consequently, the mis‐stimulation was significantly reduced compared with the LSE. In the case of an interference stimulus, a highly complex expression is possible with a nonlinear equation of the reference electric field of each frequency of each electrode as a variable (Huang & Parra, 2019; Rampersad et al., 2019). When the depth increases, there are more non‐target regions at a closer distance to the target; therefore, the concentration performance of tTIS also decreases, as with single‐frequency stimulation. However, it always performs better than tACS.

To date, tES has not been attempted because of its poor accuracy in deep regions, but in invasive deep brain stimulation (Montgomery Jr & Baker, 2000; Perlmutter & Mink, 2006), multiple stimulations have been attempted to simultaneously stimulate two or more deep regions (Parker et al., 2020; Slotty et al., 2015; Stefani et al., 2009). If elaborate stimulation of the deep area is possible and optimizable with tTIS, tES can also attempt multi‐stimulation in deep regions. We found that optimizing a single‐frequency stimulation using neural networks was noticeably better than the optimization results of LSE. Although LSE finds an answer that minimizes the least squares error from the ideal target, tACS has inherent difficulty approaching the ideal solutions and the results of the objective function set to evaluate successful stimuli are poor. Conversely, USNN optimizes in the direction of concentrating the stimulus of the target and reducing the magnitude and area of the mis‐stimulus. Consequently, the results of the visual plot of USNN are also more convincing. However, generating the strongest stimulation at the target location failed, and some mis‐stimulation remained. In the case of tTIS, the performance of the peak, concentration, and mis‐stimulation ratios was better than that of tACS. However, in the results of position 4 of the multi‐stimulation 1–4, about a quarter of them failed to provide the strongest stimulus to the target and had higher mis‐stimulation than the other multi‐region stimulation cases in tTIS. In this case, the same result was obtained even when alternative approaches were tried, such as changing the initial value or adding a hidden layer. Although stimulation with precise targeting is possible even in deep regions through interference stimulation with a nonlinear equation, we were unable to find a satisfactory solution for some targets in the solution space. When attempting multiple stimulations, this failure appeared to have a low probability for a particular combination; it was not observed in single‐location stimulations.

Interference stimulation using different frequencies has been proven to be more effective than single‐frequency tACS in stimulating the deep parts of the brain (Huang et al., 2020; Lee et al., 2020; Rampersad et al., 2019). In most tTIS studies, a two‐pair arrangement using two pairs of anodes and cathodes is frequently used. A comparison of the results of the optimization using the two‐pair electrode arrangement with those of the proposed method is shown in Figure 9. In the case of the two‐pair stimulation, it was implemented as a GA, and its solution was determined by testing many times while sufficiently converging without considering the calculation time (Appendix A). When the objective function of the GA is implemented as Equation 4, the target is visibly stimulated and is considerably concentrated in the two‐pair electrode system. However, it is not the strongest stimulation in the target region, and has a certain amount of mis‐stimulation. If a multi‐channel system can be used, the performance can be improved, and USNN can solve this problem in optimization with an extremely large number of electrodes. However, increasing the number of electrodes also causes other practical problems in addition to computational complexity. As the number of electrodes increases, it becomes difficult to implement hardware, and the problem of sensitivity to errors becomes more serious. For realistic multichannel implementation, these problems should be considered, and the electrode placement considered in a previous study using two pairs should also be considered.

The Huang method (Huang et al., 2020) maximizes the modulation of the target point by limiting the power of the non‐target and increasing the proportion of the power in the target region. When the concentration ratio is the main objective function (Appendix B), it exhibits similar results to USNN and shares the same weakness. The sum of the non‐target power is limited; however, this is focused on specific non‐target regions that are prone to stimulation. To avoid a situation in which the target is medially stimulated by focusing a stimulus on an adjacent non‐target, the proposed method considers the peak and mis‐stimulation ratios together. In terms of calculation costs, the proposed method can guarantee short calculation times owing to parallel processing via a GPU. Huang's method has the basic objective function to maximize one position within the constraint of the electrode current; however, whenever this sub‐maximization is completed, the process of checking the power constraints before calculating the slope and direction is repeated. To check the power constraint, the modulation for all positions must be calculated using Equations 1 and 3, and this process can increase the processing speed by matrix operation using a GPU, similar to USNN. Because Huang's method and the proposed method have different optimization goals and parameters that can be set for the level of precision in each method, it is difficult to fairly compare the computational efficiency of the optimization algorithm.

The optimization performance of USNN was compared with GA using the same objective function and electrode system, and in the last line of Figure 9, the visual plot of the best result and the loss according to generation is shown. Although the solution through GA is not an existing study of tTIS optimization, it has been widely used in similar problems in other fields, and a direct comparison with the proposed algorithm is possible. Even if we exclude the inefficiency of calculating 1000 losses for a population in one generation, it can be seen that the convergence speed of USNN is much faster. Even though there were 10 times more parameter updates than USNN, the performance of GA did not reach a level similar to that of USNN.

All the methods were tested under the same conditions. In each instance, the hidden layer configuration, objective function, and number of repetitions were the same. The same conditions were implemented, but this was not the best method. Iteratively checking the results and changing the parameters can lead to better results. For example, the three ratios of the objective function are multiplied by the same exponent 1 in these results; however, the exponent of the focal ratio component can be increased if the concentration is unsatisfactory to make the response of changing the concentration ratio more sensitive. Another example is that the criteria for mis‐stimulation can be relatively strict. An example of this is presented in Appendix B. The results based on the architecture of the hidden layer are presented in Appendix C. Estimating electrode currents directly without a hidden layer can result in the model falling into local minima, and the convergence speed is slow. However, even if only one hidden layer with only two nodes is added, optimization can easily result in local minima in the solution space. The time required for one iteration increases with the number of weights to be optimized; however, the optimal value is reached faster and more safely in the appropriately deep hidden layer. In a solution space with the current values of all the electrodes for each frequency as a variable, if there is no hidden layer, a simple gradient descent method is performed in the adjacent direction. By contrast, when hidden layers are used, the solution is found in a higher‐dimensional solution space with weights as variables. This enables the use of electrode currents as variables to search a much wider and denser area in the solution space.

Although simulations enable precise optimization in a short time, this study has inherent limitations associated with computational studies compared with reality. We did not consider actual hardware and experimental verification. By contrast, Song et al. in 2021 designed hardware capable of realizing multi‐channel tTIS and performed in vivo verification through c‐fos staining. However, in their case, simple optimization through the sphere model limited the study scope to the shallow brain. Computational and experimental studies should be closely linked and can complement each other's weaknesses.

For non‐invasive technology to be applied to in vivo applications, traditional tES studies, which have conducted considerable research in this area, can also provide a direction (Woods et al., 2016). Arguably, tACS is inferior to tTIS considering spatial capabilities. Although many studies have attempted to solve spatial specificity using multi‐electrodes (Datta et al., 2009; Dmochowski et al., 2011; Edwards et al., 2013), simulating only the deep region was more difficult than tTIS (Huang & Parra, 2019). However, tTIS and tACS are similar in terms of tES, except that it uses the interference of two frequencies. Additionally, tTIS is at an initial stage of research compared with investigation of tACS study, not considering spatial capabilities. This study also referred to previous tACS research for basic theory and implementation details. Therefore, follow‐up studies should also follow the same format. In fact, the tTIS study is at the initial level of research compared with investigation of tACS study, except for the spatial capabilities. This study was also based on the tACS researches for basic theory and implementation details, and follow‐up studies should also base on them.

For example, tACS studies that address individual differences in electrical conductivity and shunting issues can be considered. In tTIS, which is a nonlinear combination of electrode currents, current shunting can adversely affect the location of the targeting, and individual differences in electrical conductivity can also be severely affected. In the tACS field, shunting has been minimized by optimizing electrode placement (Neri et al., 2020), and the uncertainty of electrical conductivity has been analyzed through a mathematical model to apply it to the brain stimulation problem. (Saturnino, Thielscher, et al., 2019; Schmidt et al., 2015). The proposed method shows that various neural networks can be connected and combined and that multi‐objective functions can be compromised. In future studies, a network that calculates controllable shunting from the generated electrode current can be connected, and the effect of shunting can be observed during evaluation. The sensitivity of the result, based on individual difference in electrical conductivity, can also be reflected and an electrode current less affected by individual differences can be determined. A sensitivity model for electrical conductivity error may be supervised using computable clues (electric field distribution by frequency, etc.) or constructed as an unsupervised neural network based on a mathematical model.

## 5 CONCLUSIONS

We proposed a method to optimize the tTIS of a high‐resolution electrode system based on USNNs and analyzed the possibilities and performance in various cases. The proposed method shows precise and accurate performance (depth index of five, on average; PR of 1.161, CR of 9.40, and MR of 0.83 were obtained) in the tTIS optimization problem, which remains a difficult problem. The proposed method also demonstrates the viability of fast calculations. As the process of modulating oscillations in the deep brain becomes more accurate, it is expected to become the basis for more clinical testing and verification in the field of brain stimulation using electric fields.

## AUTHOR CONTRIBUTIONS

Sangkyu Bahn and Chany Lee conceived the initial idea. Sangkyu Bahn conducted all optimization and numerical simulation. Sangkyu Bahn and Bo‐Yeong Kang, designed the neural network. Sangkyu Bahn and Chany Lee created the models and the software for the finite element method. All authors revised and approved the final manuscript.

## CONFLICT OF INTEREST

The authors declare that no conflicts of interest exist.

## Figures and Tables

**FIGURE A1:**
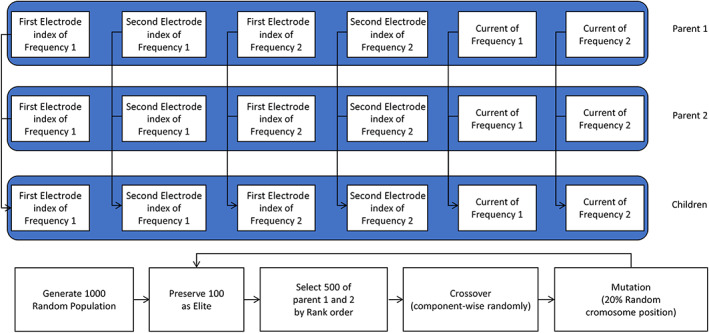
Chromosome flow chart of the genetic algorithm for two‐pair interference stimulation

**FIGURE B1:**
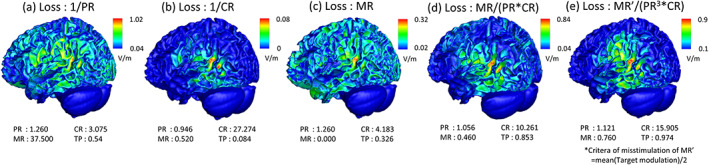
Optimization results according to the loss functions

**FIGURE C1:**
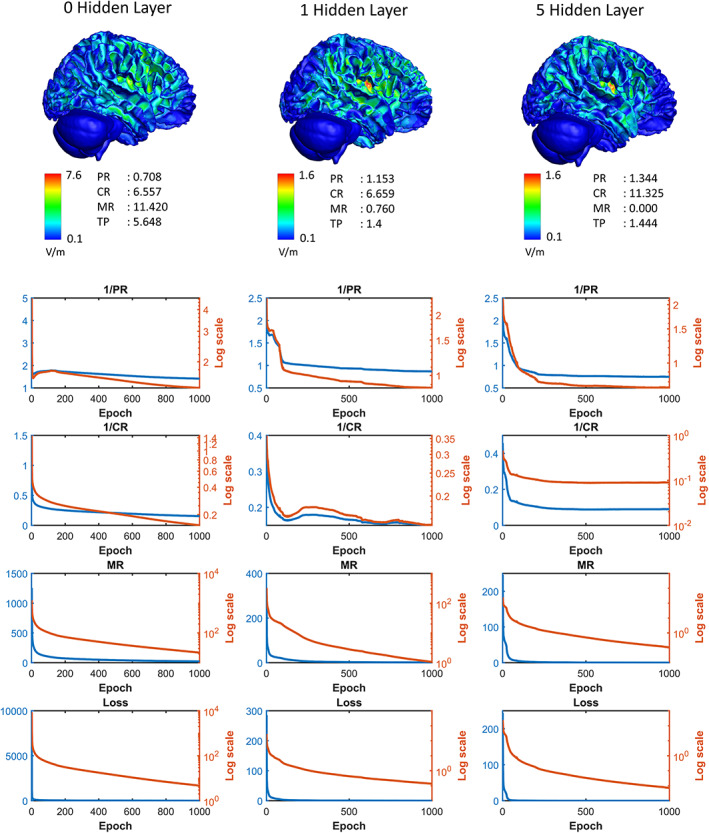
Optimization results according to change in hidden layers. The graph on the left shows 1/peak ratio, 1/focal ratio, mis‐stimulation ratio, and the product of three items for the epoch. When only one hidden layer is used, it is composed of 2 nodes, and when using 5 hidden layers, 2, 4, 8, 16, 32 nodes are configured to increase exponentially

## Data Availability

**Data:** Eleven MRIs were downloaded from HCP Archive of Human Connectome Project (https://ida.loni.usc.edu/login.jsp?page=HOME&subPage=OVERVIEW_HM&project=HCP). We downloaded ICBM data whose subjects were named as “UTHC_XXXX.” Five MRIs were downloaded from The McConnel Brain Imaging Centre (https://www.bic.mni.mcgill.ca/ServicesAtlases/NIHPDobj1). We used asymmetric templates. All processed data can be made available upon reasonable request to the corresponding author. **Code:** The processing codes can be made available upon reasonable request to the corresponding author.

## APPENDIX A GENETIC ALGORITHM FOR TWO‐PAIR tTIS

The electrode value optimization of the two‐pair interference stimulus was performed using a genetic algorithm. The composition of the chromosome consists of six terms as (electrode position by frequency) and (electrode intensity by frequency), as shown in Figure A1. The electrode position has an integer value of 1 to 69. The electrode strength has a real value of −1 to 1 and is then normalized in order for the sum of the injected currents to be 4 mA.

The initial population starts with 1000 randomly generated individuals, preserving 100 elites per generation. Here, the evaluation value uses the same function as the loss function of the proposed neural network. Based on the rank, 500 Parent 1 and Parent 2 are selected, and crossover is carried out. The crossover generates two offspring that inherit the items of Parent 1 and Parent 2 randomly for each chromosome position. Rank‐based selection and random position crossover are intended to slow the convergence speed so that it can converge to the correct answer slowly, avoiding the local minima as much as possible without being constrained by the calculation time. The mutation changes the positions corresponding to 20% of all chromosome positions of the crossed children to random values.

When these 1000 generations were repeated 100 times, 17 results converged to the best value among the test results.

## APPENDIX B PERFORMANCE ACCORDING TO THE LOSS FUNCTION

The loss function of the main test used in this study is expressed as the product of the peak, focal, and mis‐stimulation ratios. The results of using only each component as a loss function are shown in Figure B1a–c.

In the case of the peak ratio, because it is a value of one position representing a divided region, it cannot always guarantee a continuous and differentiable solution space. The peak ratio has reached a fairly high place, but the peak ratio is rather low compared to when the mis‐stimulation is a loss function, and it can no longer go to a high place. This component only guides the result so that the target can be stimulated the most strongly in the early or final fine‐tuning stage. Nevertheless, when only the peak ratio is used as the loss function, the peak ratio has a high value. However, large and small mis‐stimuli spread throughout the brain.

When only the mis‐stimulation ratio is used as the loss function, a relatively strong modulation is generated at the target position, and all modulations elsewhere are smaller than the average of the target. However, weak modulation is spread throughout the brain, and the peak value of the target is low compared with other results.

When only the focal ratio is used as the loss function, the entire modulation generated is concentrated near the target. However, the results showed that the other areas next to the target were stimulated more strongly than the target.

When using the product of the three items, each item yields slightly worse, but overall compromises. The strongest stimulus was generated in the target, the overall modulation was relatively concentrated toward the target area, and the mis‐stimulation was smaller than the area of the target. In addition, the target peak value was higher than the other results because avoiding mis‐stimulation is possible while being focused.

After checking the results, it can be adjusted according to the purpose by modifying the loss function. A simple example is presented in Figure B1. Because the concentration of the result of producing three items equally was disappointing, the exponent of the focal ratio was set to 3 to make the reaction very sensitive to the concentration ratio. In addition, to reduce the size of the part that is mis‐stimulated on the side of the target, the criteria for mis‐stimulation were lowered by half. The results showed that modulation was more concentrated than the previous results, and the size of the lateral mis‐stimulation was reduced.

## APPENDIX C PERFORMANCE ACCORDING TO HIDDEN LAYERS

If the electrode currents are directly determined using the gradient descent method without using a hidden layer, the solution easily falls into the local minima. However, by adding a hidden layer of Relu consisting of only two nodes, the network can avoid these local minima and find a better solution. As in the architecture used in the main results of this study, if five layers are configured with an exponentially increasing number of nodes, such as 2, 4, 8, 16, 32, it converges to a better result much faster. Figure C1 shows the convergence of the loss component according to the number of hidden layers in the insula region mentioned in the text.
